# Association between inflammatory cytokines and long-term adverse outcomes in acute coronary syndromes: A systematic review

**DOI:** 10.1016/j.heliyon.2020.e03704

**Published:** 2020-04-07

**Authors:** Gisela A. Kristono, Ana S. Holley, Prashant Lakshman, Morgane M. Brunton-O'Sullivan, Scott A. Harding, Peter D. Larsen

**Affiliations:** a Department of Surgery and Anaesthesia, University of Otago Wellington, New Zealand; b Wellington Cardiovascular Research Group, New Zealand; c Cardiology Department, Capital and Coast District Health Board, New Zealand

**Keywords:** Clinical research, Cardiology, Inflammation, Health sciences, Biomarkers, Outcomes, Acute coronary syndromes, Cytokines

## Abstract

**Background:**

Inflammatory cytokines are involved in the pathophysiology of acute coronary syndromes (ACS) and have been associated with major adverse cardiovascular events (MACE). We systematically reviewed studies investigating the ability of multiple cytokines to predict MACE in ACS patients with follow-up of at least one year.

**Methods:**

A Medical Subject Heading search criteria was applied on Ovid Medline(R), EMBASE, EMBASE Classic and Cochrane Library to systematically identify relevant studies published between 1945 and 2017 that had an observational study design or were randomised controlled trials. Studies were excluded if only one cytokine was analysed, follow-up period was less than one year, subjects were non-human, or blood samples were taken more than 10 days from symptom onset.

**Results:**

Ten observational studies met the inclusion criteria. Six had acceptable internal validity when evaluated for quality. The studies were varied in terms of study methods (time of blood collection, study population, cytokines assessed, MACE definition, follow-up length) and result reporting, so a meta-analysis could not be conducted. Six of the studies found significant associations between individual cytokines and MACE. Four studies measured the combined effects of multiple cytokines to predict MACE, and all had statistically significant results.

**Conclusion:**

A combination of multiple cytokines had a better association with MACE than individual cytokines. It appears promising for future studies to determine the optimal multi-marker methodology and confirm its predictive value.

## 1 Introduction

Cardiovascular disease is the primary cause of mortality worldwide [1]. Approximately half of those deaths are attributed to acute coronary syndromes (ACS), which encompasses acute myocardial infarction (AMI) and unstable angina (UA) [2]. ACS is associated with significant morbidity and financial burden, as readmission to hospital occurs in 20% of ACS patients within one year [1, 2]. Inflammation plays a pivotal role not only in the progression of atherosclerosis [3], but also in mediating removal of necrotic tissue following myocardial infarction and in shaping the repair processes that are essential for resolution of the AMI [4]. For this reason, there has been considerable interest in measuring markers of inflammation in ACS and their value in predicting major adverse cardiac events (MACE) such as death, recurrent myocardial infarction (MI), stent thrombosis, heart failure (HF) and recurrent angina [5, 6].

The most widely studied biomarker of inflammation is C-reactive protein (CRP) [6, 7]. While numerous studies have reported an association between CRP and MACE, the relationship is not sufficiently predictive for measurement of CRP to be recommended by current guidelines [8]. A large number of studies have also examined the relationship between circulating levels of individual cytokines measured after the onset of ACS and MACE, partly due to the fact that cytokines have a more direct relationship with atherosclerosis than CRP [9]. Therefore, cytokines may be better markers to investigate than CRP.

Inflammation is a complex network response of multiple different cell types to an injury, such as AMI, that involves an altered expression of cell surface markers and secretion of a large numbers of cytokines and chemokines [4]. Therefore, it is likely that measurement of a non-specific, single marker to characterise “inflammation” in this complex setting is an over-simplified approach. A chronic HF cohort study found assessment of multiple inflammatory biomarkers to be a stronger predictor of the long-term risk of adverse events when compared to a single marker approach [10]. This has also been reported in other disease states such as colorectal cancer and hepatocellular carcinoma [11, 12].

Therefore, this systematic review aims to investigate whether, in a population of ACS patients with multiple cytokines measured, characterisation of inflammation using combined cytokines analyses as opposed to a single marker approach was superior for predicting MACE.

## 2 Methods

### 2.1 Search strategy

We searched online for publications using Ovid Medline(R), EMBASE, EMBASE Classic, and Cochrane Library databases. Results found on Medline were from 1946 to 31 December 2017 and the Medical Subject Heading (MeSH) terms and keywords used were: (“myocardial infarction/ or non-ST elevation myocardial infarction/ or ST elevation myocardial infarction/” OR “acute myocardial infarction.mp.” OR “AMI.mp.”) AND (“exp Cytokines/” OR “cytokine∗.mp.”) AND (“Prognosis/” OR “prognos∗.mp.” OR “Risk Assessment/” OR “risk stratification∗.mp.” OR “predict∗.mp.”). Results were limited to those in the English language. Publications found on EMBASE and EMBASE Classic were from 1947 to 31 December 2017. Similar MeSH terms and keywords were used, but additional limitations for article-in-press status, EMBASE status or in-process status were included along with English language. Results found on Cochrane Library were from 1945 to 31 December 2017. Similar keywords and MeSH terms were used as with Medline, but the search strategy was limited to the following collections: Cochrane Reviews, Trials, Clinical Answers, Editorials and Special Collections.

### 2.2 Inclusion and exclusion criteria

All results were imported into EndNote X7 (Clarivate Analytics, PA, USA) and assessed for eligibility. Studies were included if they met the following criteria: 1) baseline blood samples collected within 10 days from symptom onset and results conducted for an ACS-only cohort; 2) at least two inflammatory cytokines or chemokines were measured and associated with MACE and had at least one year of follow-up; and 3) the study design was either an observational study, randomised controlled trial or systematic review. Cytokines and chemokines were defined as proteins released by one cell to act on another cell, either to aid in communication, chemotactic activity, or have another effect [13], and MACE was defined as a composite of any of the following: death, recurrent MI, stroke, stent thrombosis, revascularisation, recurrent UA, or HF. Studies that measured cytokines *in vitro* were excluded.

### 2.3 Quality assessment and data extraction

Titles, abstracts and keywords were first screened to assess for eligibility. The full text of all potentially eligible studies were then assessed using a screening form adapted from Boland et al [14]. The form used to assess the eligibility of the studies can be found in the supplementary material (Figure S1). Studies that were confirmed to meet the eligibility criteria were independently evaluated for quality and bias by two investigators using cohort and case-control checklists adapted from the Scottish Intercollegiate Guidelines Network (SIGN) [15]. Where there was disagreement, this was resolved by consensus decision. Study characteristics and outcomes were also collected from the full text and supplementary documents.

Univariate and multivariate analyses of the association between cytokines and MACE were extracted from the studies. Seven corresponding authors were emailed for missing data, of which one was able to provide the data required and two responded but were unable to provide the missing data. Heterogeneity of the studies, in terms of study methods and reporting methods for the results, was assessed to determine feasibility of a meta-analysis.

## 3 Results

### 3.1 Study characteristics

**Figure 1:**
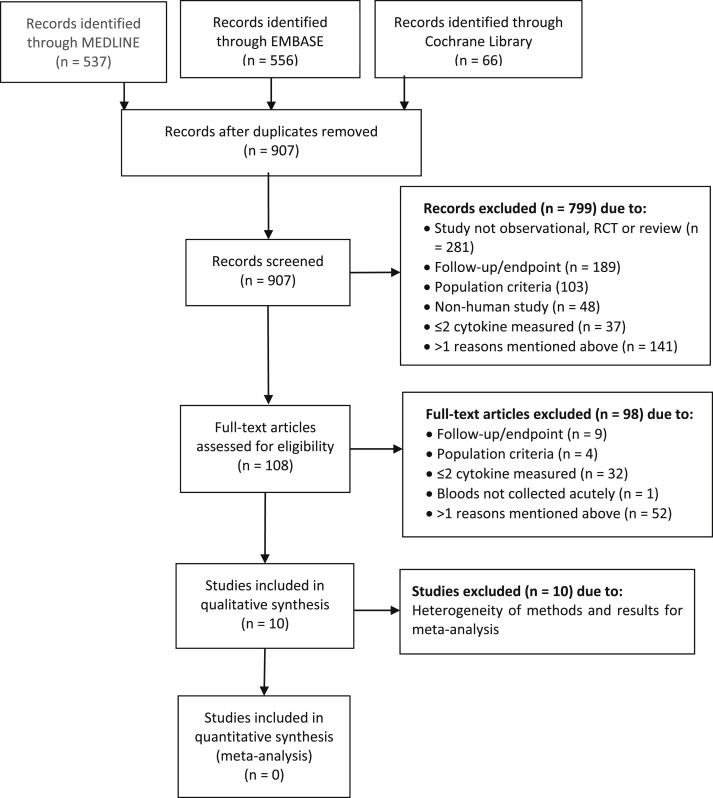
PRISMA flow diagram of selection process [26]. RCT = randomised controlled trial.

The search strategy resulted in 1159 records, of which 252 were duplicates. From the 907 abstracts screened, 108 full-text articles were assessed for eligibility, and 10 observational studies with a combined population of 3,287 ACS patients were found to meet the inclusion criteria (Figure 1) [16, 17, 18, 19, 20, 21, 22, 23, 24, 25]. There were no randomised controlled trials. There was heterogeneity between the studies in terms of the populations studied, cytokines analysed, clinical endpoints, and length of follow-up (Table 1). The variation in the definitions of MACE used and length of follow-up between the studies resulted in quite different rates of MACE between the studies. For example, Skau et al., looked at only all-cause death as the outcome, but still had a high mortality rate of 24%. This was largely due to the long period of follow-up (median 6.9 years) [16]. Conversely, Kilic et al., had a MACE rate of 29% within one year of follow-up, but their definition of MACE was a composite of cardiac death, non-fatal MI, and recurrent angina requiring hospitalisation [22]. While Novo et al., had the highest MACE rate of 67%, as they used a broad definition of MACE that included death, MI, recurrent angina, repeat revascularisation and HF and had a follow-up period of six years [17].

**Table 1:** Study characteristics.

Author (Year)	ACS Population; % MACE	Blood Collection & Medium	Cytokines/Chemokines	Detection Assay	Endpoints	Follow-up	Data Sources
Skau E, et al. (2018) [16]^a^	AMI ± invasive procedure (n = 847); 24%	<72h from admission, plasma	13 cytokines & chemokines, including IL-6, IL-18, & MCP-1	PEA chip	All-cause death	Median 6.9 years	Medical records & patient reporting
Novo G, et al. (2015) [17]^a^	AMI ± invasive procedure (n = 33); 67%	<24h from symptom onset, serum	23 cytokines, including IL-6, IL-10, MCP-1, & TNFα	Multiplex	Composite of recurrent angina, MI, death, new revascularisation, & HF	6 years	Not stated
Yu CW, et al. (2013) [18]^b^	STEMI ± invasive procedure (n = 40); 30%	<6h from symptom onset, plasma	IL-6, IL-10	ELISA	Composite of all-cause death, MI, stroke (unspecified) & TLR	4 years	Not stated
Simon T, et al. (2013) [19]^a^	AMI ± invasive procedure (n = 981); 18%	<48h from symptom onset, serum	IL-6, IL-17	Flow cytometry∗	Composite of all-cause death & MI	2 years	Not stated
Kaski JC, et al. (2010) [20]^a^	NSTE-ACS ± invasive procedure (n = 610); 9.8%	<48h from symptom onset, serum	IL-6, IL-10, IL-18	ELISA	Composite of all-cause death, MI, UA, PCI & CABG, & composite of death & MI	1 year	Outpatient clinics
Chalikias GK, et al. (2007) [21]^a^	ACS ± invasive procedure (n = 186); 26%	<1h from admission, serum	IL-10, IL-18	ELISA	Cardiac death, MI, UA requiring meds &/or urgent revascularisation	Median 15 months	Outpatient clinics, telephone & hospital records
Kilic T, et al. (2006) [22]^a^	NSTE-ACS + invasive procedure (n = 80); 29%	On admission, serum	IL-6, IL-1β, IL-10 & TNFα	ELISA	Composite of cardiac death, new-onset MI & recurrent rest angina	1 year	Not stated
Hung MJ, et al. (2006) [23]^b^	ACS + angiogram (n = 92); 21% all-cause death, 7.6% cardiac death, 0% MI, 11% recurrent angina	Before angiography, after overnight fasting, serum	IL-6, MCP-1	ELISA	Death (classified into cardiac & non-cardiac), MI and recurrent angina	Median 28 months	Hospital records, telephone & follow-up clinics
Valgimigli M, et al. (2005) [24]^b^	AMI ± invasive procedure (n = 184); 18%	14 ± 9h after symptom onset, serum	IL-6, IL-10, IL-1ra & TNFα	ELISA	Composite of all-cause death & HF	Median 406 days	Outpatient clinics & telephone
Ueland T, et al. (2005) [25]^b^	AMI + acute HF (n = 234); % not stated	<10 days of symptom onset [27], plasma	IL-6, IL-10, MCP-1	ELISA	Cardiac death, all-cause death, MI & angina	2 years	Not stated

ACS = acute coronary syndrome; AMI = acute myocardial infarction; ELISA = enzyme-linked immunosorbent assay; h = hours; HF = heart failure; IL = interleukin e.g. IL-1ra = interleukin-1 receptor antagonist, IL-1β = interleukin-1 beta; MACE = major adverse cardiovascular event; MCP-1 = monocyte chemoattractant protein-1; MI = myocardial infarction; NSTE-ACS = non-ST elevation acute coronary syndrome; PEA = proximity extension assay; STEMI = ST elevation myocardial infarction; TLR = target lesion revascularisation; TNFα = tumour necrosis factor alpha.

∗ Detection assay only for IL-17; assay for IL-6 not stated.

a Study is a cohort.

b study is a case-control.

In total, there were 25 different cytokines analysed by the 10 studies, with nine of them measuring interleukin-6 (IL-6) [16, 17, 18, 19, 20, 22, 23, 24, 25], seven measuring IL-10 [17, 18, 20, 21, 22, 24, 25], four measuring monocyte chemoattractant protein-1 (MCP-1) [16, 17, 23, 25], and three measuring interleukin-1 receptor antagonist (IL-1ra) [16, 17, 24], IL-18 [16, 20, 21], and tumour necrosis factor alpha (TNFα) [17, 22, 24]. All 10 studies were prospective, of which six were cohort and four case-control. The size of the ACS group in each study ranged from 33 to 981, and follow-up length ranged from one to 6.9 years. Due to the significant heterogeneity of the studies, a meta-analysis could not be conducted.

### 3.2 Methodological quality

The evaluation of the quality of the studies have been summarised in the supplementary material (Tables S1 and S2). Internal validity was determined by the risk of bias and completeness of adjustments for confounding. Six papers met an acceptable standard of internal validity and four were of poor quality. None of the studies had high internal validity, because no case-control study was found to be of high quality, and by definition, according to SIGN, no single cohort study design should be scored as high quality since they have an increased risk for bias [15]. We also believe that overall, none of the studies included had robust methodology. The majority of the papers did not clearly state the rationale as to why certain cytokines were chosen in their study over other cytokines. The exception being the study performed by Simon et al., who explained their interest in investigating the association between markers related to the IL-17 pathway and MACE [19]. However, six studies alluded that their rationales for choosing cytokines were based on previous studies or availability of commercial kits for analysing combinations of cytokines in a relatively inexpensive and efficient manner [16, 17, 21, 22, 24, 25]. Only one study validated their cohort size using a power calculation for one of the biomarkers they analysed, but found that their cohort may have still been underpowered for the other biomarkers [20]. Only one study mentioned a participation rate [16], and only two studies blinded their researchers from MACE outcomes when measuring cytokine levels or vice versa [19, 20].

### 3.3 MACE outcomes

Six of the studies found a significant association between individual cytokines and MACE, either by univariate or multivariate analysis [16, 17, 18, 19, 21, 24]. Four of the five studies that produced a hazard ratio (HR) for IL-6 had values above 1.00 [16, 18, 19, 20, 24], and three of those HRs were statistically significant [16, 19, 24], indicating that IL-6 may be a risk factor for MACE. There were mixed findings for IL-10, with 50% of the studies showing that the biomarker was protective for MACE [18, 20, 21]. Kaski et al. found IL-18 to be a risk factor for MACE (defined as a composite of death, MI, UA, percutaneous coronary intervention and coronary artery bypass graft) but the same cytokine had a HR below 1.00 for death and MI alone (the secondary endpoint) [20]. The other two studies that assessed IL-18 found an odds ratio (OR) and an HR per unit change above 1.00 [16, 21]. Table 2 summarises the statistically significant findings found for the clinical endpoints of the studies.

**Table 2:** Significant outcomes.

Author (Year)	Endpoints	Univariate Analysis	Adjusted Multivariate Analysis		Combined Cytokine Analyses
		Primary Endpoint	Primary Endpoint	Factors Adjusted For	
Skau E, et al. (2017) [16]	All-cause death	Results not given	IL-1ra = 1.36 (1.21–1.52)^1^ IL-6 = 1.31 (1.17–1.47)^1^ IL-8 = 1.48 (1.34–1.64)^1^ IL-16 = 1.35 (1.19–1.54)^1^ IL-18 = 1.27 (1.13–1.43)^1^ IL27A = 1.66 (1.48–1.85)^1^ MCP-1 = 1.20 (1.07–1.36)^1^ MIP-1α = 1.55 (1.39–1.72)^1^ MIP-1β = 1.24 (1.12–1.38)^1^ MIP-3α = 1.41 (1.28–1.56)^1^ CXCL16 = 1.30 (1.15–1.47)^1^	Age and sex	Penalised regression analysis showed that 32 markers (incl. IL27A, MIP-3α & CXCL16) and GDF-15 + TRAIL-R2 alone had ROC AUCs of 0.85. In combination with traditional risk factors, the AUC was 0.89
Novo G, et al. (2015) [17]	Composite of recurrent angina, MI, death, new revascularisation, & HF	IL-8 OR = 1.13 (1.00–1.28) IL-10 OR = 1.14 (0.99–1.30) MIP-1β OR = 1.01 (1.00–1.03)	Stated no statistically significant ORs were found	Not stated	Higher rank score (with all 27 biomarkers) was associated with MACE, F = 5.07; ROC curve analysis: Score of >13 cytokine levels above the median was a better predictor of MACE, with an AUC 0.72
Yu CW, et al. (2013) [18]	Composite of all-cause death, MI, stroke (unspecified) & TLR	IL-10 HR = 0.935 (0.902–0.969)	No multivariate analysis conducted	No multivariate analysis conducted	No combined analysis conducted
Simon T, et al. (2013) [19]	Composite of all-cause death & MI	IL-17 HR = 1.44 (1.07–1.95)	IL-17 HR = 1.40 (1.03–1.91) IL-17 = 0.88 (0.79–0.99)^1^ IL-6 = 1.20 (1.05–1.37)^1^	Sex; age; smoking; BMI; FHx of CAD; history of HTN, AMI, HF, renal failure, or DM; heart rate at admission; Killip class, LVEF; hospital management; & log CRP	No combined analysis conducted
Kaski JC, et al. (2010) [20]	*Primary endpoint:* Composite of all-cause death, MI, UA, PCI, & CABG *Secondary endpoint:* Composite of death & MI	Stated no statistically significant HRs were found	No statistically significant HRs were found	TIMI risk score, HF and previous CAD	No combined analysis conducted
Chalikias GK, et al. (2007) [21]	Composite of cardiac death, MI, UA requiring meds & urgent revascularisation	No data from univariate analysis included in paper	*Model 1:* IL-18 OR = 1.59 (1.11–2.27) *Model 3:* IL-10 OR = 0.6 (0.42–0.87)	*Model 1:* CRP, TnT, diagnosis on admission, revascularisation, β-blocker & EF<40% *Model 2:* Total cholesterol, LDL, HDL & triglycerides *Model 3:* Age, sex, DM, HTN, smoking, creatinine, LDL, HDL & prior CAD *Model 4:* β-blocker, statin, ACEi & revascularisation	Logistic regression of IL-18:IL-10 cytokine ratio: *Unadjusted:* OR = 1.91 (1.37–2.65) *Model 1:* OR = 2.31 (1.55–3.42) *Model 2:* OR = 1.86 (1.33–2.61) *Model 3:* OR = 2.33 (1.58–3.45) *Model 4:* OR = 2.09 (1.46–3.01)
Kilic T, et al. (2006) [22]	Composite of cardiac death, new-onset MI and recurrent rest angina.	No data from univariate analysis included in paper	Stated IL-1β, IL-6 and IL-10 ORs weren't significant	hsCRP	IL-6:IL-10 OR = 2.2 (1.2–3.9) Stated IL-1β:IL-10 OR wasn't significant
Hung MJ, et al. (2006) [23]	Death (classified into cardiac & non-cardiac), MI and recurrent angina.	No data from univariate analysis included in paper	Stated no independent predictors were found	Smoking, WBC, monocyte count, logCRP	No combined analysis conducted
Valgimigli M, et al. (2005) [24]	Composite of all-cause death & HF	TNFα HR = 1.4 (1.5–1.3)^2^ IL-6 HR = 1.16 (1.2–1.15)^2^ IL-10 HR = 1.09 (1.15–1.07)^2^	No multivariate analysis conducted	No multivariate analysis conducted	No combined analysis conducted
Ueland T, et al. (2005) [25]	Cardiac death, all-cause death, MI & angina	No non-significant RRs were stated for baseline measurements	No non-significant HRs were stated for baseline measurements	Smoking, prior MI, DM, medication, age, sex, creatinine clearance, NYHA functional class, N-BNP & hsCRP	No combined analysis conducted

All values are statistically significant (p ≤ 0.05). Values that are not statistically significant are not included. β-blocker = beta blocker; ACEi = angiotensin converting enzyme inhibitor; AMI = acute myocardial infarction; AUC = area under the curve; BMI = body mass index; CABG = coronary artery bypass graft; CAD = coronary artery disease; (hs)CRP = (high density) C-reactive protein; CXCL16 = chemokine ligand 16; DM = diabetes mellitus; EF = ejection fraction; F = analysis of variance (ANOVA) F value; FHx = family history; GDF-15 = growth differentiation factor 15; HDL = high density lipoprotein; HF = heart failure; HR = hazard ratio; HTN = hypertension; IL = interleukin e.g. IL-1ra = interleukin-1 receptor antagonist, IL-1β = interleukin-1 beta, IL-27A = interleukin-27 subunit alpha; LDL = low density lipoprotein; LVEF = left ventricular ejection fraction; MCP-1 = monocyte chemoattractant protein-1; MI = myocardial infarction; MIP-1α = macrophage inhibitory protein-1 alpha; MIP-1β = macrophage inhibitory protein-1 beta; MIP-3α = macrophage inhibitory protein-3 alpha; N-BNP = N-terminal brain natriuetic peptide; NYHA = New York Heart Association; OR = odds ratio; PCI = percutaneous coronary intervention; ROC = receiver operator characteristic; RR = relative risk; TIMI = thrombolysis in myocardial infarction; TLR = target lesion revascularisation; TNFα = tumour necrosis factor alpha; TnT = troponin T; TRAIL-R2 = tumour necrosis factor-related apoptosis-inducing ligand receptor 2; UA = unstable angina; WBC = white blood cell count.

1 HRs calculated for per unit change.

2 HRs calculated by comparing patients with biomarker levels above the median to those with values below the median.

The eight studies that conducted a multivariate analysis made adjustments for a variety of potential confounders, based on what was found to be statistically significant in the univariate analysis. Three of the eight studies that measured individual cytokines on multivariate analysis found that a portion of those cytokines were significantly associated with MACE [16, 19, 21]. Skau et al. and Chalikias et al. used several models adjusted for different groups of confounders [16, 21]. Skau et al. had four models: one for age and sex alone; one for traditional risk factors for MACE; one for age, sex and biomarkers including growth differentiation factor-15 (GDF-15), and TRAIL receptor-2 (TRAIL-R2); and a final model including traditional risk factors and the selected biomarkers. All four models produced high area under the curves (AUCs) from receiver operator curves ranging from 0.79 for the model adjusting for only age and sex, to 0.88 for the model adjusting for both traditional risk factors and the selected biomarkers. Chalikias et al. had four models based on: clinical factors that were significant on univariate analysis, lipid-related risk factors, MACE-related risk factors and medications. Out of these four models, IL-10 and IL-18 individually were only significantly associated with MACE in one or two of these models, while a combined IL-18/IL-10 ratio was significantly associated with MACE in all models.

Four studies analysed the association between MACE and the combined effect of multiple cytokines [16, 17, 21, 22]. Skau et al. used L_1_ penalised regression analysis to determine the optimal set of cytokines needed for predicting all-cause death [16]. Initially they found that 32 biomarkers gave a receiver operating characteristic (ROC) area under the curve (AUC) of 0.85, but reducing this set of markers to only GDF-15 and TRAIL-R2 produced the same AUC. Combining these two markers with traditional risk factors for MACE in multivariate analysis resulted in a ROC AUC of 0.89, with a net reclassification improvement of 0.09 (p = 0.001). Out of the 27 cytokines analysed, Novo et al. found that an additive score of greater than 13, with a point given for each cytokine concentration above the median, resulted in an AUC of 0.72 [17]. Chalikias et al. and Kilic et al. analysed a pro-inflammatory and an anti-inflammatory cytokine as ratios (such as IL-6/IL-10) and found that they were significant predictors of MACE [21, 22]. For Chalikias et al., the ORs were greater for all four models of the ratios compared with the individual cytokines [21]. For Kilic et al., the ratio OR for IL-6/IL-10 was significant compared with the non-significant ORs for IL-6 and IL-10 individually [22].

## 4 Discussion

In the 10 studies analysed in this systematic review, substantial heterogeneity was observed in methodology including the cytokines and chemokines studied, timing of blood collection, definition of MACE, length of follow-up, and method of statistical analysis. All studies had either acceptable or poor internal validity, with most giving no clear rationale behind the choice of cytokines studied and a generally poor reporting on the validation of cohort size. However, all four of the studies that did a multi-marker analysis showed a significant statistical association with MACE.

To answer the primary study aim, a systematic review was chosen because a systematic search would ensure an inclusion of most, if not all, relevant studies. However, this study type also allowed us to compare the methodologies across studies. This review highlighted the heterogeneity in methodology for studies assessing the prognostic value of inflammatory cytokines in ACS patients with at least one year of follow-up for MACE. This heterogeneity included the cytokines selected for analysis. One of the main reasons why it was not appropriate to conduct a meta-analysis was that many of the cytokines were investigated in two studies or less. Many of the studies mentioned in their introductions that previous studies had found some or all of the cytokines of focus to be significant in ACS [17, 21, 22, 24, 25]. Investigating the reproducibility of previous findings is important, but there was no explanation as to why certain cytokines were chosen in each study over others that have also been proven to correlate with MACE following ACS. However, it is recognised that analysing all cytokines and chemokines that have been associated with ACS would be extremely time consuming and expensive to do. One study implied that the decision was made by the assay kits available [16]. It is common for limited resources to be a barrier to optimal methodologies. Only one study clearly explained their interest in exploring the role of IL-17 and other markers related to the IL-17 biological pathway with MACE [19]. With greater availabilities of affordable assay kits and further studies clarifying which markers are most promising for predicting MACE, this may lead to improved rationale for choosing which cytokines to analyse.

Cytokines investigated for its association with MACE have traditionally been measured after the onset of AMI due to accessibility of samples in this period. However, another factor that caused heterogeneity between the studies was the differences in the time after the onset of ACS at which blood samples were collected. Three of the studies acknowledged that measuring cytokine levels from only one time point could be a limitation, as the levels were dynamic within the acute phase, and the blood samples might not have been collected at the peak cytokine levels [19, 21, 23]. Skau et al. also mentioned that their study could not determine to what extent the biomarkers remained elevated in the acute phase, as they only collected one sample within 72 hours [16]. The few studies that have collected serial blood samples have consistently shown that cytokine levels fluctuate significantly in the acute phase after an AMI [28, 29, 30]. Many cytokines have not been assessed for its levels across time, so it is unknown whether some may have steady-state levels across the acute phase of AMI, and what the significance of fluctuating or steady cytokine levels may be for predicting MACE. Further research is required to investigate this. While it is relatively straightforward to collect single samples from patients at some time point during a hospital admission, the complexity of any study increases considerably if the time point is more precisely defined relative to symptom onset, or if multiple time points are selected. However, despite the increased complexity, moving away from an opportunistic sampling approach to sampling at deliberately chosen time points within the evolution of myocardial infarction may result in more consistent and more sensitive and specific results.

These 10 studies also showed variation in cohort size, ranging from 33 to 981 patients, with only one containing a power calculation to validate their cohort in their paper [20]. Power calculation is a common method to determine cohort size [31]. However, Kaski et al. found it difficult to ascertain that the cytokine chosen for the power calculation is sufficient to represent the other cytokines [20]. The rate of MACE in a study will be dependent on the risk of the population enrolled, the definition of MACE used (endpoints included in the composite) and the length of follow-up. The definition of MACE used can also lead to bias if more subjective endpoints, such as recurrent angina, are included. In this review, the MACE rates ranged from 9.8% to 67%, which is likely to reflect the differences in the factors mentioned. Statistically, a certain number of events should be added per variable included in a regression model, with this number being widely debated [32]. However, it is unlikely that Novo et al.'s study has enough events for the 46 variables included in the logistic regression analysis, with only 11 patients in the MACE group [17]. Overall, it is important that future studies address these deficiencies in methodology.

The current paradigm is that individuals with elevated levels of pro-inflammatory cytokines and low levels of anti-inflammatory cytokines are at increased risk of MACE following AMI. However, only five of the studies included in this review found a statistically significant association between specific individual cytokines and MACE via univariate or multivariate analyses (Table 2) [17, 18, 19, 21, 24]. Table 2 shows that although the majority of the point estimates for IL-10 demonstrated the opposite, some studies had point estimates that did not contribute to this conclusion. Liu et al. conducted a meta-analysis of 12 studies on IL-10 in ACS patients and found that an elevated level of IL-10 was associated with a slight increase in risk of MACE during a follow-up of at least 30 days (relative risk, RR = 1.009, 95% confidence interval 1.005–1.013, P < 0.001) [33], which is a surprising result for a cytokine traditionally thought of as anti-inflammatory. Variations in the methodologies of these studies are likely to contribute to these inconsistent findings, reflecting a need for a more robust and homogeneous methodology.

There is clear evidence that cytokines work together in a complex inflammatory network, where an imbalance of pro-inflammatory and anti-inflammatory cytokines may lead to adverse outcomes [34]. As mentioned in the Introduction, examining individual biomarkers that are non-specific to ACS may be insufficient to capture a snapshot of this inflammatory network and how it relates to MACE [35, 36]. Observations from our systematic review showed that of the four studies that conducted a combined biomarker analysis, all had statistically significant results [16, 17, 21, 22]. Therefore, a combined biomarker approach may be a better option for future studies to better reflect underlying inflammatory changes that could cause MACE. A prospective study that carefully considers the limitations of previous studies mentioned in this review into the study design is required to confirm this hypothesis.

The four studies all used different methods to combine their markers, with no obvious rationale for the method chosen in each study. The methods included creating ratios of pro-inflammatory to anti-inflammatory cytokines [22], logistic regression [17, 21], creating a rank score [17], and penalised regression analysis [16]. A limitation of these methods is that because cytokines have overlapping functions [34], there is a risk that these studies may have over-counted their effects of each cytokine by analysing each one as an independent risk factor. A complex inflammatory network cannot be represented by a simple analysis of multiple cytokines. To our knowledge, only three studies have investigated over 10 cytokines in ACS patients [16, 17, 37], with one providing no analysis between the cytokine scores and MACE after at least one year of follow-up [37]. The inflammatory cytokines included in Skau et al. and Novo et al.'s studies serve as a good foundation for examining a combined inflammatory panel to predict risk of MACE [16, 17], but further investigation is needed to determine which set of cytokines creates the optimal inflammatory panel and how best to combine these markers into a composite score.

The initial promise of targeting inflammation as a therapeutic intervention has not yet led to improved clinical outcomes [38, 39]. However, the idea that those with pathologically elevated inflammation might be effectively targeted with anti-inflammatory therapy has received new impetus due to the results of the CANTOS trial [40]. Improved methodologies to characterise pathological inflammation in both the acute and chronic stages of myocardial infarction may allow this promise to be achieved to a greater completion. We would suggest that a multi-marker approach is sufficiently promising to warrant further investigation, despite the limitations discussed above in the existing literature.

The selection process required to answer our systematic review question caused our literature search to be limited to papers looking specifically at combined versus single marker measures and comparing them in the paper analyses. This represents potential selection bias in Table 2, as the small number of single cytokine results may not be representative of the totality of the literature. As mentioned earlier, one of the limitations of this study was being unable to conduct a meta-analysis, largely due to the heterogeneity of the methodologies. Statistically, it would also be incorrect to combine the different summary statistics, such as HRs and ORs, for meta-analysis [41]. This reflects a limitation in the current literature. This review has only focussed on the prognostic ability of cytokines and chemokines, but other inflammatory biomarkers may also be important to consider, such as white blood cell subtypes [42, 43]. We were also interested in long-term outcomes of at least one year, as it would benefit clinically to predict MACE using cytokines as a secondary prevention method. However, this caused a large number of studies to be excluded from our review. Lastly, we chose to include studies that had ACS populations, i.e. UA as well as AMI, to be able to have a sufficient number of studies in this review.

## 5 Conclusion

Although some studies have reported significant associations between individual cytokines and MACE, we found mixed associations from the 10 studies included in this review. However, a combined analysis of multiple cytokines may have greater association with MACE. This review highlights some gaps in the current body of evidence on the relationship between inflammatory cytokines and MACE in ACS patients, showing that there is considerable heterogeneity in methods and results, such as cytokine selection, blood collection times and cohort sizes. We would recommend future studies to provide a rationale for their cytokine selection and be adequately powered to detect a clinically significant difference in appropriately defined MACE outcomes. Further studies are also required to determine the importance of time of blood collection. New, robustly designed, prospective studies that address the specific deficits of past studies, are required to test whether a multi-marker approach may be a better option. Further investigation is required for which set of markers creates an optimal panel and which method is most accurate for combining the markers.

## Declarations

### Author contribution statement

Gisela A. Kristono: Conceived and designed the experiments; Performed the experiments; Analyzed and interpreted the data; Contributed reagents, materials, analysis tools or data; Wrote the paper.

Ana S. Holley, Peter D. Larsen: Conceived and designed the experiments; Analyzed and interpreted the data; Contributed reagents, materials, analysis tools or data; Wrote the paper.

Prashant Lakshman, Morgane M. Brunton-O'Sullivan: Performed the experiments; Wrote the paper.

Scott A. Harding: Conceived and designed the experiments; Wrote the paper.

### Funding statement

This work was supported by University of Otago Doctoral scholarships awarded to Gisela Kristono and Morgane Brunton-O'Sullivan. Dr Ana Holley was funded by Division of Health Sciences University of Otago Postdoctoral Fellowship.

### Competing interest statement

The authors declare no conflict of interest.

### Additional information

No additional information is available for this paper.

## Appendix A Supplementary data

20200326 Supplementary Material Kristono G.docx 20200326 Supplementary Material Kristono G.docx
