# Supplementary material for: Association between inflammatory cytokines and long-term adverse outcomes in acute coronary syndromes: A systematic review
Source: Heliyon. 2020 Apr 7;6(4):e03704. doi: 10.1016/j.heliyon.2020.e03704 (PMC7138910; doi:10.1016/j.heliyon.2020.e03704)
Supplement: 20200326 Supplementary Material Kristono G.docx [file mmc1.docx]

**Figure S1 Eligibility screening form for full papers**

| Review Question: How well are inflammatory cytokines that are acutely measured in acute coronary syndrome (ACS) patients predictive of major adverse cardiovascular events (MACE) during a follow-up of at least one year? | | | |
| --- | --- | --- | --- |
| Inclusion Criteria (based on PICOS framework):  Population = Adult patients with baseline blood collection that occurred acutely (≤10 days) after onset of ACS  Intervention = At least two inflammatory cytokines (defined as a protein that is produced by an immune cell and acts on another cell) measured from the blood samples  Comparator = Can have a control group etc., but results that we are interested in must only come from an ACS-only group  Outcomes = MACE outcomes (death, recurrent myocardial infarction, heart failure, ischaemic stroke, stent thrombosis, revascularisation, recurrent unstable angina) with at least one year follow-up  Study design = Observational case-control or randomised controlled trial (RCT) | | | |
| PROGNOSTIC ABILITY OF CYTOKINES IN ACS PATIENTS SCREENING AND SELECTION TOOL | | | |
| Reviewer Name: | | **Date:** | |
| Author Name: | | **Year:** | |
| Title: | | **Journal:** | |
| Patient Population  Interventions  Outcomes  Study Design  Overall decision | **Include**  Human adults  ACS patients  Baseline bloods collected ≤10 days from symptom onset  If RCT, baseline bloods collected before initiation of treatment  Has at least two inflammatory cytokines analysed in the lab  Correlates baseline cytokine levels with MACE  At least one year follow-up  RCT  Case-control  **INCLUDED** | | **Exclude**  Non-human subjects  Children  Did not have results looking at an ACS-only group  Baseline bloods not collected acutely, after treatment, or before occurrence of ACS  Has one or no inflammatory cytokines assessed from the baseline blood samples, or methods not included  No follow-up/follow-up not one year  Looked at other outcomes  Did not correlate outcomes with baseline cytokine levels  Review, meta-analysis, editorial, letter, conference abstract, cohort, cross-sectional, etc.  **EXCLUDED** |
| Notes: | | | |

Form adapted from Boland et al. [14]

1. Cohort quality assessment

| **Quality Factor** | **Skau E, et al. (2017) [17]** | **Novo G, et al. (2015) [18]** | **Simon T, et al. (2013) [20]** | **Kaski JC, et al. (2010) [21]** | **Chalikias GK, et al. (2007) [22]** | **Kilic T, et al. (2006) [23]** |
| --- | --- | --- | --- | --- | --- | --- |
| 1.1 Appropriate and focussed study question | Y | Y | Y | Y | Y | Y |
| 1.2 Groups selected from comparable source populations | NA | NA | NA | NA | NA | NA |
| 1.3 Participation rate (%) | 94.4 | CS | CS | CS | CS | CS |
| 1.4 Subjects having outcome during enrolment | NA | NA | NA | NA | NA | NA |
| 1.5 Lost to follow-up (%) | 0.24 | CS | <2 | 10 | 2.6 | CS |
| 1.6 Comparison between full participants and lost to follow-up | NA | N | CS | Y | CS | N |
| 1.7 Defined outcomes | Y | Y | Y | Y | Y | Y |
| 1.8 Outcome assessment made blind to cytokine measurements | N | N | Y | Y | N | N |
| 1.9 Recognition that knowledge of cytokine levels could influence outcome assessment | N | N | Y | Y | N | N |
| 1.10 Methodology of cytokine measurement | Y | Y | Y | Y | Y | Y |
| 1.11 Methodology of outcome assessment | Y | Y | Y | Y | Y | Y |
| 1.12 Repeated cytokine measurements | CS | CS | CS | CS | CS | CS |
| 1.13 Adjustment for confounding | Y | Y | Y | Y | Y | Y |
| 1.14 Confidence intervals provided | Y | Y | Y | Y | Y | Y |
| 2.1 Internal validity of study^‡^ | + | + | + | 0 | + | 0 |
| 2.2 Association between cytokines and outcome | CS | CS | Y | Y | Y | CS |
| 2.3 Study results applicable to New Zealand population | Y | Y | Y | Y | Y | CS |

Y = Yes, N = No, NA = Not applicable, CS = Cannot say, ++ = High quality, + = Acceptable quality, 0 = Poor quality

**^‡^** Internal validity assessed by risk of bias and completeness of adjustments made for potential confounders

Adapted from the Scottish Intercollegiate Guidelines Network checklists [15]

1. Case-control quality assessment

| **Quality Factor** | **Yu CW, et al. (2013) [19]** | **Hung MJ, et al. (2006) [24]** | **Valgimigli M, et al. (2005) [25]** | **Ueland T, et al. (2005) [26]** |
| --- | --- | --- | --- | --- |
| 1.1 Appropriate and focussed study question | Y | Y | Y | Y |
| 1.2 Cases and controls selected from comparable source populations | Y | Y | Y | CS |
| 1.3 Same exclusion criteria for cases and controls | CS | Y | Y | CS |
| 1.4 Participation rate per group (%) | CS | CS | CS | CS |
| 1.5 Baseline comparison between cases and controls | Y | Y | Y | N |
| 1.6 Cases defined and differentiated from controls | Y | Y | Y | Y |
| 1.7 Controls established as non-cases | Y | Y | Y | Y |
| 1.8 Measures to prevent knowledge of primary exposure influencing case ascertainment | NA | N | N | N |
| 1.9 Methodology for cytokine and outcome measurement | Y | Y | Y | Y |
| 1.10 Adjustment for confounding | Y | Y | Y | Y |
| 1.11 Confidence intervals provided | Y | Y | Y | Y |
| 2.1 Internal validity of study | + | 0 | + | 0 |
| 2.2 Association between cytokines and outcome | CS | CS | Y | CS |
| 2.3 Study results applicable to New Zealand population | CS | CS | Y | CS |

Y = Yes, N = No, NA = Not applicable, CS = Cannot say, ++ = High quality, + = Acceptable quality, 0 = Poor quality

**^‡^** Internal validity assessed by risk of bias and completeness of adjustments made for potential confounders

Adapted from the Scottish Intercollegiate Guidelines Network checklist [15]
